# Investigation of Whole Blood Thiamine Concentration in Independently Ambulatory Residents of a Provincial Town in Japan: A Cross-Sectional Study

**DOI:** 10.7759/cureus.38800

**Published:** 2023-05-09

**Authors:** Nozomu Uchida, Mayumi Ishida, Akira Yoshioka, Takao Takahashi, Daisuke Furuya, Yasuhiro Ebihara, Hiroshi Ito, Akiko Yanagi, Hideki Onishi, Izumi Sato

**Affiliations:** 1 Department of General Medicine, Ogano Town Central Hospital, Ogano, JPN; 2 Department of Psycho-oncology, Saitama Medical University International Medical Center, Hidaka, JPN; 3 Department of Clinical Oncology, Mitsubishi Kyoto Hospital, Kyoto, JPN; 4 Department of Supportive Medicine, Saitama Medical University International Medical Center, Hidaka, JPN; 5 Department of General Medicine, Saitama Medical University International Medical Center, Hidaka, JPN; 6 Department of Laboratory Medicine, Saitama Medical University International Medical Center, Hidaka, JPN; 7 Department of General Medicine, Ito Internal Medicine and Pediatric Clinic, Fukuoka, JPN; 8 Department of Nursing, Maruyama Memorial General Hospital, Iwatsuki, JPN; 9 Department of Clinical Epidemiology, Graduate School of Biomedical Sciences, Nagasaki University, Nagasaki, JPN

**Keywords:** provincial town, korsakoff syndrome, wernicke encephalopathy, thiamine deficiency, thiamine

## Abstract

Background

Thiamine deficiency (TD) is an important public health problem in nutrition, occurring in 2-6% of the population in Europe and the US, whereas thiamine levels are reported to be significantly reduced by 36.6-40% in some populations of East Asia. However, there is little information available at present, regarding factors such as age, despite the continued aging of society. Further, studies such as those mentioned above have not yet been undertaken in Japan, the country in which population aging is most advanced.

Objective

To investigate TD in the Japanese community-dwelling individuals who are independently ambulatory.

Methods

We undertook an examination of TD in blood samples obtained from 270 citizens in a provincial town, aged 25-97 years, who were able to walk to the venue and provide informed consent for inclusion in this research and of whom 8.9% had a history of cancer. We summarized the demographic characteristics of the subjects. The whole-blood thiamine concentrations were measured using the high-performance liquid chromatography method. A value of 21.3 ng/ml or less was taken as low and a borderline value was set as less than 28 ng/ml.

Results

The mean (±SD) whole blood thiamine concentration was 47.6 ± 8.7 ng/ml. No TD was observed to exist participating in this study, with no subjects even showing show borderline values. Further, there was no significant difference in thiamine level between those aged 65 or older and those aged less than 65.

Conclusions

No cases of TD were observed among the subjects in this study, nor was the concentration of thiamine found to be related to age. It is possible that the frequency of TD might be very low in citizens who have a certain level of activity. In the future, it is necessary to expand the prevalence of TD to a wider range of subjects.

## Introduction

Thiamine, in its biologically active form thiamine pyrophosphate, is an essential coenzyme for glucose metabolism [1]. However, as humans cannot synthesize thiamine, intake from an external source is necessary [2]. In addition, insufficient thiamine intake that continues for at least a few weeks results in thiamine deficiency (TD) as thiamine can only be stored in the body for approximately 18 days [2].

Such a deficiency may lead to Wernicke encephalopathy (WE), the classic triad of signs of which are impaired consciousness, ataxia, and nystagmus; however, all three signs are present in less than 20% of cases [3]. Due to the variety of neuropsychiatric symptoms, patients often go untreated for WE [4,5]. WE can be resolved without complications by early treatment, while a delay in treatment can result in severe and irreversible damage to the brain, with 85% of those untreated progressing to Korsakoff syndrome (KS), which has a mortality rate reported to be close to 20% [2]. Therefore, early detection and treatment are vital.

TD remains a current condition of concern and the frequency of TD has been reported to be associated with various pathological conditions and in various regions. In terms of pathological conditions, reports of TD in cancer patients are increasing, mainly in Europe and the United States. TD was observed in 55% of patients receiving psycho-oncological care, 45% of patients diagnosed with delirium in psychiatric departments, and in 33% of those receiving chemotherapy. Results from a palliative care unit reported that 28% of patients had low TD while 36% were borderline.

It is also a report of TD as a cause of worsening psychiatric symptoms in patients with dementia and the recognition of thiamine deficiency in the elderly is an important clinical issue.

A recent regional study of TD found that 35% of Gambian women of reproductive age were deficient, indicating that TD was a major problem. In Europe and the US, the frequency has been reported to be 2-6.4% [6,7]. However, among aging East Asian countries, it is reported to be much higher, with 36.6% of the elderly urban Indonesian population [8] and 40% of surveyed mainly non-elderly adults in the Seoul metropolitan area in South Korea [9] experiencing TD. In addition, despite the continued aging of society, no survey on thiamine deficiency and age has been conducted.　

In recent years, the measurement of thiamine by the high-performance liquid chromatography (HPLC) method has provided accurate data on thiamine concentrations in living organisms [10].

As a result, TD in association with various pathologies and conditions other than alcoholism has been clarified. Clinically, TD has also been reported in cancer patients [11-13], depressed patients [14], and residents at various facilities [15,16]. In addition, patients with asymptomatic TD have been reported [11,17] and require careful attention.

Measurement of the blood thiamine concentration in the general population and confirmation of the current status can allow the prevalence of TD and its background factors to be clarified, thereby aiding the early detection and treatment of TD.

As mentioned above, various findings on TD have been collected, but there are no reports on TD at the regional level in Japan. Information on TD in Japan, in which the aging of society is the most advanced in the world, can be academically valuable when considering the future conditions facing other countries. To investigate TD in the general Japanese population, we explored TD by measuring blood samples among community-dwelling individuals who are independently ambulatory as a first step.

## Materials and methods

Methods

This is a cross-sectional study conducted in Ogano town on October 28th, 2018. Ogano Town, located in a mountainous region about 80Km northwest of Tokyo, has a population of about 11,500 (approximately 37% of who are aged 65 years or over). A health festival is organized annually for the town residents. Thus, we advertised this study in the town newspaper twice in advance and recruited participants on the day of the festival.

This is almost similar to the method of collecting subjects in a Korean study [9]. Eligibility criteria included the following: (1) Ogano town residents aged 18 years and above, (2) Independently ambulatory, (3) Japanese-speaking.

We excluded subjects who were taking any supplements, including thiamine, those who did not understand the purpose of the research, those hospitalized, in care or aged facility, or at-home care, and those who could not attend the examination site. The sample size (n = 262) was calculated with a confidence level of 95%, a sample error of 3 ng/ml, and a standard deviation of the mean whole blood thiamine concentration of 24.8 ng/ml, as reported in a previous study.

After obtaining written informed consent, we collected the self-administered questionnaire from the subjects. The survey was conducted by pre-trained staff to provide standardized support. The self-administered questionnaire was originally made by UN, MI and HO based on previously published papers [1,11]. It included the following items: date of birth, gender, height, weight, medical history (cancer, stroke, heart disease, diabetes, hypertension, hyperlipidemia, kidney disease, liver disease, depression, dementia), lifestyle choices (alcohol intake; type/amount), smoking status (Brinkman index), decreased appetite over the previous 2 weeks (if yes, half of usual or less than 1/3 of usual), sleep problems, and vitamin usage.

Lastly, we collected venous blood samples from study subjects. The measurement of whole blood thiamine concentration was entrusted to BML Inc., Shibuya-ku, Tokyo, Japan, by means of HPLC (reference range: 21.3-81.9 ng/ml). This method is precise, rapid, and less susceptible to factors that alter enzyme activity, thus providing a more sensitive assay for screening cases of TD in a clinical setting and for research purposes [18].

The lower limit of the normal range observed by the testing company is 21.9, but it has been pointed out that there are patients with borderline values and there is also a report that 28 ng/ml or more is the normal thiamine level based on the analysis of 602 cases in Japan. A value of less than 28 ng/ml was, therefore, set as a borderline value.

Statistical analysis

We summarized the demographic and lifestyle data using the raw data and percentages (%) for categorical variables and mean (standard deviations {SD}) for continuous variables. We created a histogram of whole blood thiamine concentration in the study subjects and calculated whole blood thiamine concentration means for the following categories and drew box plots: sex, age (< 65 years/≥ 65 years), history of hypertension, and BMI (< 20/≥ 20). We categorized BMI according to the criteria for malnutrition (BMI under 20) of Health Japan 21 established by the Japanese Ministry of Health, Labour, and Welfare. Health Japan 21 is a ten-year plan including specific goals for promoting the health of the nation.

We used SAS statistical software version 9.4 for Windows (SAS Institute Inc., Cary, NC, USA) for all analyses. This study was approved by the Ethics Committee of Ogano Town Central Hospital (Ogano No. 13) and the Ethics Committee of Saitama Medical University International Medical Center (Application No. 18-121).

## Results

A total of 270 subjects were included in this study, which accounts for 2.7% of all residents and 4% of residents aged 65 years and over. No subjects were unable to answer the questionnaire.

The mean (±SD) age was 65.5 ± 12.41 years (≥ 65 years, 64%), with 74.4% of the subjects being female. The mean (±SD) BMI was 22.9 (3.36) (≥ 20, 82.2%). With regard to medical history, 2.2% had experienced or were undergoing treatment for stroke, 7.0% heart disease, 3.0% kidney disease, 1.9% liver disease, 8.9% diabetes, 28.2% hypertension, 8.1% hyperlipidemia, 0.7% hyperuricemia, 1.1% were receiving cancer treatment, 7.8% had recovered from cancer (Table 1).

**Table 1:** Characteristics of Study Participants BMI: body mass index

Variable		N(%)
Female		201(74.4)
Age (years)		
	Mean ± SD	65.5 ± 12.41
	Min-Max	25-97
	< 65	96(35.6)
	≥65	174(64.4)
BMI*		
	Mean ± SD	22.9 ± 3.36
	Min-Max	15.6-33.5
	<20	48(17.8)
	≥20	222(82.2)
History of diseases		
	Hypertension	76(28.2)
	Diabetes mellitus	24(8.9)
	Cancer	24(8.9)
	Hyperlipidaemia	22(8.1)
	Heart disease	19(7.0)
	Kidney disease	8(3.0)
	Stroke	6(2.2)
	Liver disease	5(1.9)
	Hyperuricemia	2(0.7)

Only 11.1% of the subjects were living alone. Appetite was about 50% that of usual and less than 33% that of usual in 0.7% of respondents each. We found that 35.6% of respondents experienced insomnia on occasion and 7.4% always. The average alcohol consumption was 2.6 drinks/week (Table 2).

**Table 2:** Life style 1 Drink is equivalent to 10 g of alcohol.

Variable		N(%)
Living alone		30(11.1)
Appetite		
	as usual	266(98.5)
	half of usual	2(0.7)
	less than 1/3 of usual	2(0.7)
Sleep problems		
	no	154(57.0)
	sometimes	96(35.6)
	always	20(7.4)
Alcohol consumption		
	Yes	78(28.9)
Alcohol consumption (drinks/week) ※		
	Mean ± SD	12.7 ± 13.2
	Median	9.1
	Min-Max	0.65-75.6
Smoking status		
	Current smokers	33(12.2)
	Formar smokers	13(4.8)
	Never smokers	224(83.0)
Brinkman index		
	Mean ± SD	514.7 ± 394.2
	Median	445.0
	Min-Max	20.0-1880

**Figure 1:**
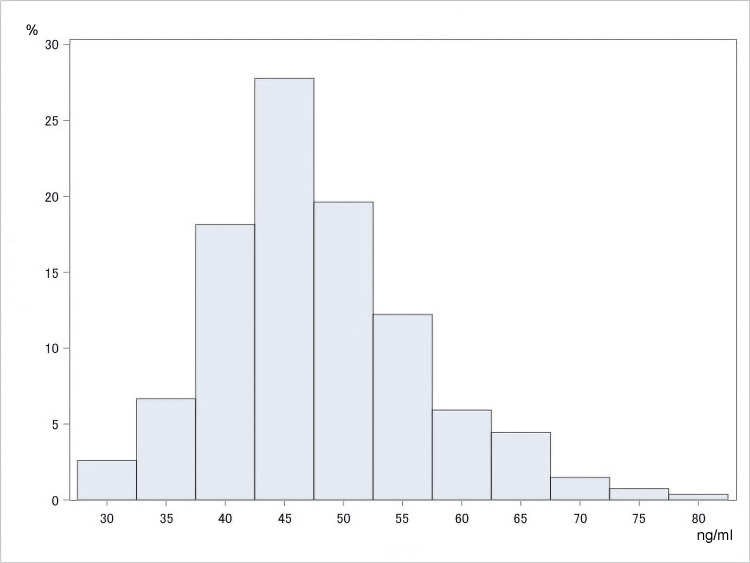
Histogram of whole blood thiamine concentration among the total population (n=270)

**Figure 2:**
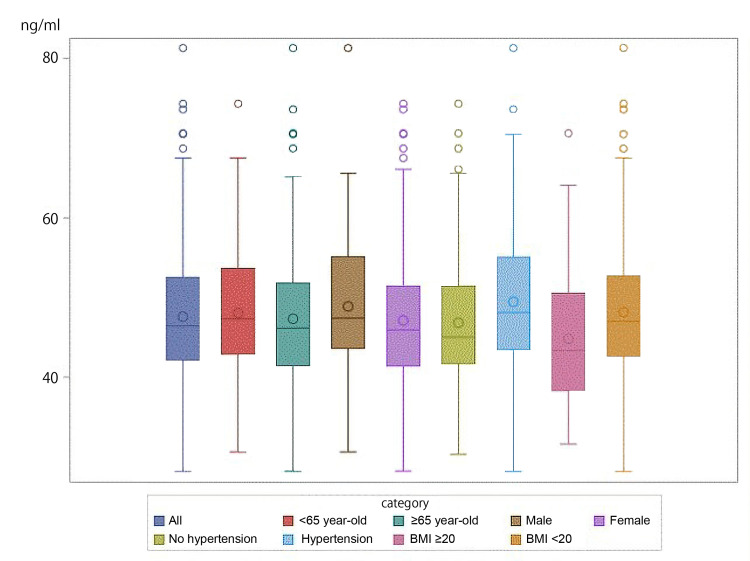
Box plots of whole blood thiamine concentration by age (≥65 years/< 65 years), gender, presence/absence of hypertension, and BMI (≥20/ <20)

No cases of TD (< 21.3 ng/ml) were observed in this study, and no cases were observed to show a borderline value (less than 28 ng/ml). The mean (±SD) whole blood thiamine concentration was 47.57 ± 8.73 ng/ml (male, 48.87 {± 9.02} ng/ml; female, 47.12 {± 8.60} ng/ml; < 65 years, 48.03 {± 8.47} ng/ml; ≥ 65 years, 47.31 {± 8.88} ng/ml; no hypertension, 46.83 ± {8.32} ng/ml; hypertension, 49.45 {± 9.58} ng/ml; BMI < 20, 44.81 {± 8.47} ng/ml; BMI ≥ 20, 48.16 {± 8.68} ng/ml) (Table 3, Figures 1, 2). There was no significant difference in thiamine level between those aged 65 years or older and those aged less than 65 years. 

**Table 3:** Whole blood thiamine concentration

Category	n	Mean ± SD (ng/ml)	Min-Max (ng/ml)
Total	270	47.6 ± 8.73	28.2-81.3
Male	69	48.9 ± 9.02	30.6-81.3
Female	201	47.1 ± 8.60	28.2-74.3
<65 years	96	48.0 ± 8.47	30.6-74.3
≥65 years	174	47.3 ± 8.88	28.2-81.3
No hypertension	194	46.8 ± 8.32	30.3-74.3
Hypertension	76	49.5 ± 9.48	28.2-81.3
BMI* <20	48	44.8 ± 8.47	31.6-70.6
BMI* ≥20	222	48.2 ± 8.68	28.2-81.3
*BMI, body mass index			

## Discussion

As a first step in investigating TD in the general population, we undertook an examination of TD and its background factors in citizens who participated in this study in a provincial town based on the measurement of blood samples using the HPLC method. As a result, it was found that there were no cases of TD among the 270 people surveyed, with none of the patients showing even a borderline value.

Several factors contributed to this result. In this study group, the mean amount of alcohol consumed was extremely low at 2.5 drinks/week (1 drink is equivalent to 10 g of pure alcohol), and 71.1% (n = 192) of the participants did not drink alcohol at all. Case reports on patients with WE reported that they consumed 56 to 84 standard drinks/week of alcohol [19,20]. Our survey found no residents within that range. Thus, the absence of TD in the study may be associated with a large number of non-drinkers and an overall low level of alcohol consumption.

A systematic review of micronutrient intake in community-dwelling older adults suggests that about half of men and 39% of women may not have adequate thiamine intake [21]. It has also been reported that the serum concentration of thiamine is reduced in the elderly [22,23]. Further, a survey by the Ministry of Health, Labor and Welfare revealed that many Japanese people did not meet the recommended intake [24]. However, our study did not show a significant difference in whole blood thiamine concentration by age (< 65 or ≥65 years) or sex. This discrepancy may be related to the fact that only 1.5% (n = 4) of the study population reported a decrease in appetite.　

In addition, TD is known to exist in patients with heart disease [25], kidney disease [26], and cancer [11,27-29], but the number of subjects with an underlying disease in this study was small. Therefore, we compared the whole blood concentrations of thiamine in the participants with/without hypertension, which was the most common underlying disease; however, no significant difference in values was observed. With regard to hypertension, a correlation between TD and pulmonary hypertension has been reported [30]; however, to our knowledge, there have been no reports to date on essential hypertension and TD. Based on a report that weight loss is a risk factor for TD [11], subjects were divided by BMI (<20 or ≥20) and the serum levels of thiamine in the two were measured. Again, no significant difference was observed between the two groups.

The results of this study differ from those from other East Asian populations, such as Indonesia and South Korea. These differences may be related to differences in the measurement method applied in each study, but future studies are needed to clarify the reason for the large discrepancy.

Several limitations to this study should be acknowledged. First, the target group was not a random sample of residents of the town but a convenience sample of residents who were able to provide informed consent for inclusion in this study. Thus, no patients who were hospitalized, in care or aged facility, or at-home care were included in the study. In addition, the fact that they cooperated in giving blood samples themselves suggests that the subjects were interested in their own health. This suggests the presence of selection bias. The number of participants who actually had an underlying illness was also small. In addition, no information on the drugs administered was available. In other words, we were unable to examine whether the drugs administered may have affected thiamine metabolism.

The results of this study suggest that independently ambulatory community-dwelling residents, such as those in our study population, do not have a thiamine deficiency, regardless of sex, age, BMI, or whether they live alone or not. This study will be useful as basic data for the assessment of TD in the adult Japanese population. In the future, it is necessary to conduct surveys to identify populations that are likely to be thiamine deficient and to explore the risk factors.

## Conclusions

No cases of TD or of subjects with borderline thiamine values were observed in this community, including among those with underlying illnesses and lifestyle-related diseases but who were sufficiently active and were able to understand the intent of such a study. There was no significant difference in thiamine level between those aged 65 years or older and those aged less than 65 years. Further studies are expected to reveal the level of TD in the general population.
